# Job Satisfaction and Associated Factors among Health Professionals Working at Public and Private Hospitals in Bahir Dar City, Northwest Ethiopia: A Comparative Cross-Sectional Study

**DOI:** 10.1155/2021/6632585

**Published:** 2021-08-26

**Authors:** Amare Geta, Gashaw Andargie Biks, Endalkachew Dellie, Lake Yazachew

**Affiliations:** ^1^Bahir Dar City Administration, Amhara National Regional State, Bahir Dar, Ethiopia; ^2^Department of Health Systems and Policy, Institute of Public Health, College of Medicine and Health Sciences, University of Gondar, Gondar, Ethiopia

## Abstract

**Introduction:**

Job satisfaction is a pleasurable or positive emotional state resulting from evaluating one's job or job experiences. However, knowledge of workplace factors that either satisfy employees to keep working or dissatisfy them making them leave their jobs or working places is essential for decision-making. Thus, this study is aimed at assessing job satisfaction and associated factors among healthcare professionals working at public and private hospitals in Bahir Dar city, northwest Ethiopia.

**Methods:**

An institution-based comparative cross-sectional study design was conducted from 10 February 2020 to 29 May 2020. A total of 520 health professionals were selected from public and private health facilities using stratified systematic random sampling techniques. Data were collected using structured pretested self-administered questionnaires. A binary logistic regression model with Huber-White robust standard error was fitted to identify job satisfaction and associated factors among healthcare professionals working at public and private hospitals. A less than 0.05 *p* value and an Adjusted Odds Ratio (AOR) with 95% Confidence Interval (CI) were considered to have a statistically significant association with the outcome variable.

**Results:**

The overall magnitude of job satisfaction was 55.2% (95% CI: 51.0, 59.4%). At public and private hospitals, the provider's satisfaction was 29.0% (95% CI: 23.2, 35.1%) and 81.23% (95% CI: 76.6, 85.8%), respectively. Working at private hospital (AOR: 8.89; 95% CI: 5.14, 15.35), pleasant nature of work (AOR:  1.82; 95% CI: 1.05, 3.15), autonomy (AOR: 2.37; 95% CI: 1.29, 4.33), adequate supportive supervision (AOR: 2.42; 95% CI: 1.33, 4.40), good reward and recognition (AOR: 3.04; 95% CI: 1.37, 6.75), and high normative commitment (AOR: 2.57; 95% CI: 1.48, 4.43) were factors affecting the overall job satisfaction of health professionals.

**Conclusions:**

The magnitude of job satisfaction was relatively low in private and public hospital health professionals and severe among health professionals working in public hospitals. Healthcare policy-makers and hospital managers need to develop and institutionalize evidence-based satisfaction strategies considering the predictors of health professional's job satisfaction.

## 1. Introduction

The Ethiopian healthcare system promotes public-private partnerships to ensure equity and quality of healthcare delivery. The public sector is composed of government health organizations that primarily serve the poor segment of the population. In contrast, the private sector is composed of private health facilities for-profit and serves the population segment who can afford out-of-pocket payment [1]. To ensure equity and quality of healthcare provided, availability and accessibility of highly committed and well-performing healthcare professionals are paramount [1–3]. Consequently, healthcare professionals' job satisfaction is a fundamental issue in improving access and quality healthcare for the population [4]. Job satisfaction is a pleasurable or positive emotional state resulting from evaluating one's job or job experiences [3, 5].

The job satisfaction of healthcare professionals is highly important in building up employee interest and efficiency. Higher job satisfaction determines better employee performance and a higher level of patient satisfaction ultimately to gain competitive advantage and greater productivity of the organization [4, 6]. Researchers found that employees satisfied with their job prefer to stay with their employers [7].

However, various findings showed that job satisfaction among health workers is low [8, 9]. In Asian countries, various cross-sectional studies reported the level of job satisfaction as Vietnam 41.8% [10], Pakistan 41% [11], Nepal 76% [12], and Sri Lanka 23.7% [13]. Similarly, studies in Africa reported the peak level of job satisfaction owned by Nigeria (90.4%) [14] and the least being in Ghana (36.4%) [15]. In Ethiopia, numerous studies showed that the magnitude of job satisfaction stretched from 41.46% in western Ethiopia to 60.3% in Jimma [6, 16–21].

Several studies showed a significant and positive relationship between demographic factors such as age, working experience, profession, level of education, and gross monthly salary with job satisfaction [4, 22]. According to the level of education, job satisfaction levels of healthcare staff with high school and technical school degrees were lower than healthcare staff with university degrees and master's and above degrees. Specialist doctors, practitioner doctors, and health officers tended to show greater job satisfaction than nurses [23].

According to different studies, opportunities for reward, nature of work, supervision, relations, contact with colleagues, training, and benefit status of the organization were factors affecting employees' job satisfaction [8, 24, 25]. Employees feel satisfied in organizations that allow them to participate in decision-making processes, improve their skills and knowledge, and enhance work autonomy [3], whereas too many working hours, administrative burdens, heavy workload, lack of time, poor performance evaluation system, and lack of recognition were factors identified as decreasing job satisfaction [26–28].

Employee job satisfaction is also influenced by working space and responsibility, opportunity to develop, staff relations [4, 29], quality of the working environment, and organizational commitment [30]. In particular, a study done in the United States hypothesized that “organizational commitment is directly related to job satisfaction,” and the study results confirmed that job satisfaction was strongly interrelated and associated with organizational commitment in a study done in Iran [31, 32].

Similarly, previous researchers found that health workers in Ethiopia tend to be unsatisfied with many aspects of their job, for instance, training opportunities, decision-making autonomy, poor working environment, and their chances of promotion [33–36].

Furthermore, there are limited studies on job satisfaction and associated factors in the study area as to our search. Besides, no comparative study is conducted at all in the study area.

Therefore, this study is aimed at assessing job satisfaction and associated factors among healthcare professionals working at public and private hospitals in Bahir Dar city, northwest Ethiopia.

Thus, this study will let managers better understand the underlying issues to implement strategies to improve health organization policy and principles.

## 2. Methods

### 2.1. Study Design and Settings

A facility-based comparative cross-sectional study was conducted from 10 February 2020 to 29 May 2020 to assess job satisfaction and associated factors among healthcare professionals working at public and private hospitals in Bahir Dar city. Bahir Dar city is the Amhara National Regional State's capital city, Ethiopia, located 565 km from Addis Ababa to the northwest. Based on Ethiopia's 2007 Central Statistical Agency (CSA) report, Bahir Dar city had 339683 [37]. The city had been divided into six subcities and 24 kebeles (the smallest administrative units in Ethiopia). The city has three public hospitals, ten public health centers, four private hospitals, and thirty private clinics. According to the Amhara National Regional State health bureau performance report of 2019, there were 1653 and 504 health professionals in public and private hospitals in Bahir Dar city who provide services [37]. Information obtained from these hospitals' administrative offices provides different outpatient, inpatient, and operation room theatre services.

## 3. Population and Sampling

This study's source population was all health professionals working at public and private hospitals in Bahir Dar city. Likewise, all health professionals who were working for at least six months at public and private hospitals in Bahir Dar city during the study period were the study populations. Contract and voluntary employees were excluded from the study.

The sample size for the proportion of job satisfaction was determined using a double population proportion formula. An assumption of 95% confidence level, 80% power, P1 (proportion of patient satisfaction at public hospitals) 31.7% [25], P2 (proportion of patient satisfaction at a private hospital) 50%, and 10% nonresponse rate was considered. The final sample size for n1 (for public hospitals) = 271 and n2 (for private hospitals) = 271 yielded a total of 542.

The participants were identified by using a systematic sampling technique. The list of health workers was taken from payroll in the human resource management department.

### 3.1. Variables and Measurement

#### 3.1.1. Health Professional

A health professional is anyone who has earned a diploma and/or bachelor's degree or higher in health science from an accredited college or university.

The dependent variable for this study was job satisfaction. It was measured by a five-point Likert scale (1: very dissatisfied to 5: very satisfied) of 20 items. Respondents who scored 60% and above the sum of the satisfaction scales were considered satisfied [38].

#### 3.1.2. Organizational Commitment

Organization commitment is a health professional's degree of attachment towards their employer. Three dimension scales measured it; a score with more than 60% of the commitment scales' sum represented a high organizational commitment [39].

Affective commitment is a health professional's attitude regarding the alignment of personal and organizational goals. This was measured by using three items, and a 5-point Likert scale scored each item from one, denoting “strongly disagree,” to five “strongly agree.”

#### 3.1.3. Continuance Commitment

Continuance commitment is a health professional's desire to stay with the organization in light of costs associated with leaving (i.e., seniority and pension plans). This was measured by using three items, and each was scored on a 5-point Likert scale with one denoting “strongly disagree” and five representing “strongly agree.”

#### 3.1.4. Normative Commitment

Normative commitment is a health professional's decision to stay with an organization because he or she feels obligated. This was measured using three items; each was scored on a 5-point Likert scale, with one denoting strongly disagree and five strongly agree.

#### 3.1.5. Work Environment

Work environment describes the quality of the working environment, both its physical attributes and the degree to which it provides meaningful work. It was measured by using four items; each was scored on a 5-point Likert scale.

#### 3.1.6. Supervisor Support

Supervisor support describes the supervisors' responsibility both in preventing and in solving employee problems. It was measured by using ten items, each scored on a 5-point Likert scale.

#### 3.1.7. Recognition and Reward

Recognition and reward describe employee perception about the way they are encouraged. It was measured by 5-point Likert scale questions with one denoted very disagree and five very agree.

#### 3.1.8. Coworker Relationship

Coworker relationship describes the participants' interpersonal relationships within their workplace. It was measured by using four items, each scored on a 5-point Likert scale.

#### 3.1.9. Pay and Benefit

Pay and benefit describe employees' expectations of fairness and adequate compensation on a day's pay for a day's work. It was measured by using four items, each scored on a 5-point Likert scale.

#### 3.1.10. Educational Opportunity

Educational opportunity describes the availability of advancement opportunities for employees within the organization. It was measured by using four items, each scored on a 5-point Likert scale.

#### 3.1.11. Organizational Policy

The organizational policy describes the way how an organization implements policy and strategy. It was measured by using four items, each scored on a 5-point Likert scale.

#### 3.1.12. Autonomy

Autonomy reflects participants' autonomy in initiating and continuing their work behaviors and processes, such as making decisions about work methods, pace, and effort. It was measured by using two items, each scored on a 5-point Likert scale.

#### 3.1.13. Performance Appraisal

Performance appraisal describes the participants feeling on the measurement of their actual performance. It was measured by using three items, each scored on a 5-point Likert scale.

#### 3.1.14. Nature of Work

Nature of work describes the type of work the participants do (variety, meaningfulness). It was measured using nine items each scored on a 5-point Likert scale.

#### 3.1.15. Workload

Workload describes the participant's work requirement, the amount of time, and resources for this requirement. It was measured by using six items, each scored on a 5-point Likert scale.

### 3.2. Data Collection Tools and Procedure

Data were collected using a Minnesota Satisfaction Questionnaire (MSQ) and a pretested and structured self-administered questionnaire and adopted from previously published literature [38]. The questionnaire was prepared in English and translated to Amharic, then returned to English to check its consistency. The tool's reliability for each subscale was checked using Cronbach's alpha reliability test with a score of greater than 0.83.

To ensure the data quality, two diploma nurses and one BSc public health professional were recruited as data collectors and supervisors, respectively. Also, training regarding the study objectives and data collection process was given to data collectors and supervisors for one day. Moreover, the questionnaire was pretested among 5% of the sample size in Gondar Referral Hospital. Participants were informed that the codes were used to facilitate tracking of the completeness of their respective questionnaires. The supervisors and the principal investigator were responsible for checking on the completeness of the data on-site. Incomplete questionnaires were put in offices arranged for this purpose so that participants completed their questionnaires. Furthermore, intensive supervision was done by the supervisors and principal investigators throughout the data collection period. All participants were acknowledged for their time and assistance.

### 3.3. Data Processing and Analysis

After the data was checked for its consistency and completeness, data were entered into EpiData version 4.6 and exported to SPSS version 20 for further analysis. Descriptive analysis was done for each variable in the study by running frequencies and percentages. Tables and charts were used for presenting results to give a clear picture of the magnitude and relationships of various study variables. Binary logistic regression and multiple logistic regression analyses were used to determine the significant association between the independent and dependent variables. In the logistic regression model, an estimator with the Huber-White robust standard errors was used. Variables with a *p* value of less than 0.2 in the binary logistic regression analysis were candidates for multiple logistic regression analysis. Association between the independent and dependent variables was considered significant when the *p* value was less than 0.05 from multiple logistic regression analysis.

## 4. Results

### 4.1. Sociodemographic Characteristics of the Respondents

A total of 520 study participants were involved in this study, with a response rate of 95.9%. Two hundred fifty-nine participants with a response rate of 95.9% were from the public hospitals, and 261 respondents with a 96.3% response rate were from the private hospitals. The median age of the participants was 26 (IQR = 25-31) years. Half of the respondents were male, 260 (50%), and most of the respondents were married 318 (61.2%). Three hundred nineteen (61.3%) of the respondents had a bachelor's degree, and two hundred forty-six (47.3%) of the respondents had 1-5 years of work experience. The median monthly salary of the respondents was 5294 (IQR = 2300-22180) Ethiopian birr. Around 423 (81.3%) and 493 (94.8%) of respondents were Orthodox Christian followers and Amhara in their ethnicity, respectively.

The majority of respondents were male, 132 (51%), and more than half of the respondents were married 173 (66.8%). One hundred seventy-nine (69%) of the respondents had a bachelor's degree, and one hundred ten (42.5%) of the respondents had 6-10 years of work experience. The respondents' median monthly salary was 5294 Ethiopian birr with an interquartile range (2411-13140). The median age (IQR) of the study participants was 27 (24-30) years, ranging from 21 to 50 years, and the greatest number of study participants, 183 (70.1%), is under the age category between 20 and 29 years. The majority of respondents were female 133 (51%), and more than half of the respondents were married 145 (55.6%). One hundred forty (53.6%) of the respondents had a bachelor's degree, and one hundred forty-seven (56.3%) of the respondents had 1-5 years of work experience. The respondents' median monthly salary was 4646 Ethiopian birr with an interquartile range (3500-6700) and ranges from 2300 to 22180 (Table 1).

### 4.2. Organizational and Job-Related Characteristics of Respondents

Overall, 51.0% and 59.6% of respondents worked in a pleasant and safe working environment, respectively. Around 48.5% and 60.8% of respondents got adequate supportive supervision and freedom on their jobs, respectively. Only 21.6% and 37.5% of the participants were given a clear job description and used their annual leave in public hospitals.

The majority of the respondents, 88.4% at the public and 87% at private hospitals, had a high workload. About 37.5% and 69% of public and private hospitals had got short-term training. Twelve and 17.4% of participants were satisfied with recognition, reward, and pay and benefit in public hospitals. Similarly, most of the respondents 87.7%, 63.6%, and 82.8% had good coworker relationships, adequate supportive supervision, and high affective commitment in private hospitals, respectively (Table 2).

### 4.3. Magnitude of Job Satisfaction

In this study, among job satisfaction items, the highest respondents' value was for the praise they get for doing a good job (67.1%), whereas the lowest respondents' value was for the way their coworkers get along with each other (47.9%). Healthcare professionals' overall magnitude of job satisfaction was 55.2% (95% CI: 51.0, 59.4%). However, the magnitude of job satisfaction at the public hospital was 29.0% (95% CI: 23.2, 35.1%) and at the private hospitals was 81.23% (95% CI: 76.6, 85.8%) (Table 3).

### 4.4. Factors Associated with Health Worker Job Satisfaction in Public and Private Hospitals

The multivariable logistic regression analysis, age, performance appraisal, reward and recognition, and normative commitment were significant variables for job satisfaction in public hospitals. Respondents aged greater than or equal to 30 years were 13 times more satisfied (AOR: 13.06; 95% CI: 4.83, 35.34) compared to respondents aged less than 30 years. Healthcare professionals who agree with the performance appraisal practice were 86% less likely to be satisfied than those who disagree with the performance appraisal practice (AOR: 0.24; 95% CI: 0.09, 0.63). Healthcare professionals who got good rewards and recognition were 4.09 times more likely to be satisfied compared to those who did not get good rewards and recognition (AOR: 4.9; 95% CI: 1.17, 14.29). Study participants with high normative commitment were 3.14 times more likely to be satisfied than those with low normative commitment (AOR: 3.14; 95% CI: 1.30, 7.61) (Table 4).

Job description, nature of work, and autonomy were significant variables for job satisfaction in private hospitals. Accordingly, the respondents who got clear job descriptions were 5.59 times more likely to be satisfied than their counterparts (AOR: 5.59; 95% CI: 1.28, 24.31). Healthcare professionals working in the pleasant nature of work were 71% less likely to be satisfied than those working in unpleasant work (AOR: 0.29; 95% CI: 0.10, 0.85). Healthcare professionals who had autonomy in decision-making were 3.0 times more likely to be satisfied compared to their counterparts (AOR: 3.00; 95% CI: 1.01, 8.95) (Table 4).

### 4.5. Factors Associated with Overall Health Professional Job Satisfaction

In the final multivariable logistic regression analysis model, workplace work, job description, nature of work, autonomy, supportive supervision, reward and recognition, and normative commitment were factors associated with overall job satisfaction (*p* < 0.05). Subsequently, health professionals working in private hospitals were 8.89 times more likely to be satisfied as compared to those who work in public hospitals (AOR: 8.89; 95% CI: 5.14, 15.35).

Healthcare professionals working in the pleasant nature of work were 1.82 times more likely to be satisfied than those working in the unpleasant nature of work (AOR: 1.82; 95% CI: 1.05, 3.15). Study participants who had autonomy for decision-making were 2.37 times more likely to be satisfied as compared to those who had no autonomy for decision-making (AOR: 2.37; 95% CI: 1.29, 4.33). Respondents who got adequate integrated supportive supervision were 2.42 times more likely to be satisfied with their job compared to those who did not get adequate supervision (AOR: 2.42; 95% CI: 1.33, 4.40).

Healthcare professionals who got good rewards and recognition were 3.04 times more likely to be satisfied compared to those who did not get good rewards and recognition (AOR: 3.04; 95% CI: 1.37, 6.75). Study participants with high normative commitment were 2.57 times more likely to be satisfied than those with low normative commitment (AOR: 2.57; 95% CI: 1.48, 4.43) (Table 5).

## 5. Discussion

In this study, the magnitude of overall job satisfaction was found to be 55.2 (51.0, 59.4)%. The finding showed that the health professionals working at private hospitals were more satisfied 81.2% (76.6, 85.8%) than those working at public hospitals 29 (23.2, 35.1%). This discrepancy could have resulted from differences in infrastructure in the health institutions, administrative issues, socioeconomic characteristics, and healthcare workers' organizational setup.

In this study, the magnitude of overall job satisfaction was comparable with previous studies conducted among health professionals in Ethiopia, at Addis Ababa 52.9% [17], East Gojjam zone (54.2%) [16], and northwest Ethiopia (46.9%) [20]. But this finding was lower than those of studies conducted in Jimma University Specialized Hospital 60.3% [19], Nepal (76%) [12], and Nigeria (90.4%) [14]. On the other hand, it is higher than studies done among health professionals in Ghana 36.4% [15], Vietnam 41.8% [10], Pakistan 41% [11], India 24.7% [5], Sri Lanka 23.7% [13], Harari region in Ethiopia 44.2% [6], western Ethiopia 41.46% [21], and Amhara region 46.9% [18]. The possible reasons for this variation might be due to the study time differences, differences in socioeconomic status, and differences in the study's geographical area.

In this study, the magnitude of job satisfaction at the public hospitals was lower than studies done in Vietnam, 41.8% [10]; Pakistan, 41% [11]; Ghana, 36.4% [15]; Harari region in Ethiopia, 44.2% [6]; Jimma University Specialized Hospital, 60.3% [19]; and Amhara region, 46.9% [18]. The magnitude of job satisfaction in private hospitals in this study was higher than in a study conducted in Nepal (76%) [12]. On the other hand, it was lower than a study conducted in Nigeria (90.4%) [14]. However, it is higher than studies done in Ethiopia [11, 17, 19, 20]. Possible reasons for this variation might be the differences in infrastructure in the health institutions, study area differences, and the tools used to measure the outcome variable that might affect job satisfaction. In this study, the tools used to measure the outcome variable were the Minnesota Satisfaction Questionnaire, whereas the majority of previous studies used other tools like the Job Satisfaction Survey (JSS) and Satisfaction of Employees in Healthcare (SEHC).

The odds of job satisfaction were higher among healthcare professionals who work in private hospitals. This was supported by other studies conducted in China and New York [7, 23].

Healthcare professionals working in a pleasant nature of work were more likely to be satisfied than those who are working in an unpleasant nature of work. This finding is supported by other studies carried out elsewhere [8, 40]. This can be the fact that if employees are engaged with a kind of work and they labeled it as worthwhile, are with pride, and are able to see results, it is likely that they will be satisfied with their job. Study participants who had autonomy for decision-making were more likely to be satisfied as compared to those who had no autonomy for decision-making. This finding is congruent with other studies conducted in India and Chicago [3, 26]. This can be explained as when employees had freedom of decision to accomplish their assigned task and chance to control scheduling of their work, they are likely to be satisfied.

Besides, in this study, health professionals who got adequate supportive supervision were more likely to be satisfied with their job as compared to those who did not get supportive supervision. This finding was consistent with a study from the western Amhara region [25]. These factors were opportunities for reward, nature of work, supervision, and benefit status of the organization [8]. Health professionals who got rewards and recognition were more likely to be satisfied with their job than those who did not get rewards and recognition. Also, those working in a pleasant nature of work were more satisfied than those who worked in the unpleasant nature of work. This finding was in line with a study conducted elsewhere [8]. Finally, healthcare professionals who reported a high normative commitment were more likely to be satisfied with their job than their counterparts. This was consistent with a study conducted elsewhere [41]. This was because if employees feel a sense of belongingness or are involved and linked emotionally with the organization, they are likely to be satisfied.

## 6. Strength and Limitations of the Study

The possible limitation of the study was social desirability and a recall bias which may lead to artificially inflated variables. To minimize this effect, a self-administered questionnaire was used. Besides, variables were categorized, which may probably hide information. Furthermore, this study was not triangulated with a qualitative method.

## 7. Conclusions

The magnitude of job satisfaction was relatively low in both private and public hospital health professionals and severe among health professionals working in public hospitals. This study revealed that healthcare professionals' job satisfaction was relatively low in private and public hospitals and somehow severe in the public hospitals at Bahir Dar city. The pleasant nature of work, good reward and recognition system, and high affective commitment were positively associated with healthcare professional's job satisfaction at the private hospitals. Being a medical doctor and pharmacy professional, a safe work environment, and adequate supportive supervision positively influence public hospitals' job satisfaction. In light of this finding, healthcare policy-makers and hospital administrators should consider the identified factors to improve healthcare professionals' job satisfaction in private and public hospitals.

## Figures and Tables

**Table 1:** Sociodemographic characteristics of the respondents at public and private hospitals, Bahir Dar city, Ethiopia (*n* = 520), 2020.

Variables	Public hospitals (*n* = 259) *N* (%)	Private hospitals (*n* = 261) *N* (%)	Total (*n* = 520) *N* (%)
Sex			
Male	132 (51)	128 (49)	260 (50)
Female	127 (49)	133 (51)	260 (50)
Age			
20-29	151 (58.3)	183 (70.1)	334 (64.2)
30-39	87 (33.6)	71 (27.2)	158 (30.4)
≥40	21 (8.1)	7 (2.7)	28 (5.4)
Educational level			
Diploma	73 (28.2)	97 (37.2)	170 (32.7)
Degree	179 (69.1)	140 (53.6)	319 (61.3)
Above degree	7 (2.7)	24 (9.2)	31 (6.0)
Religion			
Orthodox	216 (83.4)	207 (79.3)	423 (81.3)
Muslim	29 (11.2)	34 (13.0)	63 (12.1)
Protestant	13 (5.0)	19 (7.3)	32 (6.2)
Others	1 (0.4)	1 (0.4)	2 (0.4)
Ethnicity			
Amhara	238 (91.9)	255 (97.7)	493 (94.8)
Oromo	7 (2.7)	3 (1.1)	10 (1.9)
Tigray	8 (3.1)	1 (0.4)	9 (1.7)
Others	6 (2.3)	2 (0.8)	8 (1.6)
Marital status			
Married	173 (66.8)	145 (55.6)	318 (61.2)
Single	69 (26.6)	105 (40.2)	174 (33.5)
Divorced	15 (5.8)	10 (3.8)	25 (4.8)
Separated	1 (0.4)	0 (0.0)	1 (0.2)
Widowed	1 (0.4)	1 (0.4)	2 (0.3)
Profession			
Nurse	108 (40.7)	102 (39.1)	210 (40.4)
Midwifery	49 (18.9)	27 (30.3)	76 (14.6)
Medical doctor	36 (13.9)	49 (18.8)	85 (16.3)
Laboratory	26 (10)	32 (12.3)	58 (11.2)
Pharmacy	29 (11.2)	39 (14.9)	68 (13.1)
Others	11 (4.3)	12 (4.6)	33 (6.4)
Monthly salary			
<3653	77 (29.7)	84 (32.2)	161 (31.0)
3653-5294	75 (29.0)	89 (34.1)	164 (31.5)
5295-7111	68 (26.3)	25 (9.6)	93 (17.9)
>7111	39 (15.0)	63 (24.1)	102 (19.6)
Work experience			
<1	7 (2.7)	44 (16.9)	51 (9.8)
1-5	99 (38.2)	147 (56.3)	246 (47.3)
6-10	110 (42.5)	54 (20.7)	164 (31.5)
>10	43 (16.6)	16 (6.1)	59 (11.4)
Work place			
Public hospital	259 (100)	0 (0.0)	259 (49.8)
Private hospital	0 (0.0)	261 (100)	261 (50.2)

Others in profession: health officer, anesthesiologist, and radiographer; *N*: number. Others in ethnicity: SNNPs, Benishangul-Gumuz, and Gambella. Others in religion: Adventist, pagan.

**Table 2:** Organizational and job-related characteristics of health professionals working at public and private hospitals, Bahir Dar city, Ethiopia (*n* = 520), 2020.

Variables		Public hospitals (*n* = 259) *N* (%)	Private hospitals (*n* = 283) *N* (%)	Total (*n* = 520) *N* (%)
Job description	Yes	56 (21.6)	242 (92.7)	298 (57.3)
	No	203 (78.4)	19 (7.3)	222 (42.7)
Short-term training	Yes	97 (37.5)	180 (69.0)	277 (53.3)
	No	162 (62.5)	81 (31.0)	243 (46.7)
Annual leave	Yes	108 (41.7)	182 (69.70)	290 (55.8)
	No	151 (58.3)	79 (30.3)	230 (44.2)
Nature of work	Pleasant	87 (33.6)	178 (68.2)	265 (51.0)
	Unpleasant	172 (66.4)	83 (31.8)	255 (49.0)
Responsibility	Yes	98 (37.8)	191 (73.2)	289 (55.6)
	No	161 (62.2)	70 (26.8)	231 (44.4)
Workload	High	229 (88.4)	227 (87.0)	456 (87.7)
	Low	30 (11.6)	34 (13.0)	64 (12.3)
Work environment	Safe	112 (43.2)	198 (75.9)	310 (59.6)
	Unsafe	147 (56.8)	63 (24.1)	210 (40.4)
Coworker relationship	Good	176 (68.0)	229 (87.7)	405 (77.9)
	Poor	83 (32.0)	32 (12.3)	115 (22.1)
Autonomy	Yes	132 (51.0)	184 (70.5)	316 (60.8)
	No	127 (49.0)	77 (29.5)	204 (39.2)
Pay and benefit	Fair	44 (17.0)	28 (10.7)	72 (13.8)
	Unfair	215 (83.0)	233 (89.3)	448 (86.2)
Organizational policy and strategy	Comfortable	54 (20.8)	152 (58.2)	206 (39.6)
	Uncomfortable	205 (79.2)	109 (41.8)	314 (60.4)
Performance appraisal	Yes	56 (21.6)	42 (16.1)	98 (18.8)
	No	203 (78.4)	219 (83.9)	422 (81.2)
Recognition and reward	Yes	31 (12.0)	57 (21.8)	88 (16.9)
	No	228 (88.0)	204 (78.2)	432 (83.1)
Supportive supervision	Adequate	86 (33.2)	166 (63.6)	252 (48.5)
	Inadequate	173 (66.8)	95 (36.4)	268 (51.5)
Educational opportunity	Yes	40 (15.4)	21 (8.0)	61 (11.7)
	No	219 (84.6)	240 (92.0)	459 (88.3)
Affective commitment	High	170 (65.6)	216 (82.8)	386 (74.2)
	Low	89 (34.4)	45 (17.2)	134 (25.8)
Normative commitment	High	90 (34.7)	154 (59.0)	244 (46.9)
	Low	169 (65.3)	107 (41.0)	276 (53.1)
Continuance commitment	High	58 (22.4)	138 (52.9)	196 (37.7)
	Low	201 (77.6)	123 (47.1)	324 (62.3)

**Table 3:** Magnitude of job satisfaction among health professionals working at public and private hospitals, Bahir Dar city, Ethiopia (*n* = 520), 2020.

Job satisfaction items	VD, *n* (%)	D, *n* (%)	N, *n* (%)	S, *n* (%)	VS, *n* (%)	Satisfied, *n* (%)	Dissatisfied, *n* (%)
Being able to keep not being busy all the time	206 (39.6)	44 (8.5)	95 (18.3)	156 (30)	19 (3.7)	270 (51.9)	250 (48.1)
The chance to work alone on the job	65 (12.5)	159 (30.6)	91 (17.5)	138 (26.5)	67 (12.9)	296 (56.9)	224 (43.1)
The chance to do different things from time to time	50 (9.6)	189 (36.3)	114 (21.9)	91 (17.5)	76 (14.6)	281 (54.0)	239 (46.0)
The chance to be somebody in the community	30 (5.8)	202 (38.8)	127 (24.4)	51 (9.8)	110 (21.2)	288 (55.4)	232 (44.6)
The way my boss handles his/her workers	53 (10.2)	204 (39.2)	138 (26.5)	71 (13.7)	54 (10.4)	263 (50.6)	257 (49.4)
The competence of my supervisor in making decision	39 (7.5)	182 (35)	163 (31.3)	83 (16.0)	53 (10.2)	299 (57.5)	221 (42.5)
Being able to do things that do not go against my conscience	29 (5.6)	219 (42.1)	129 (24.8)	70 (13.5)	73 (14.0)	272 (52.3)	248 (47.7)
The way my job provides for steady employment	31 (6.0)	217 (41.7)	112 (21.5)	65 (12.5)	95 (18.3)	272 (52.3)	248 (47.7)
The chance to be responsible for the work of others	33 (6.3)	178 (34.2)	169 (32.5)	79 (15.2)	61 (11.7)	309 (59.4)	211 (40.6)
The chance to tell people what to do	31 (6.0)	232 (44.6)	123 (23.7)	48 (9.2)	86 (16.5)	257 (49.4)	263 (50.6)
The chance to do something that makes use of my abilities	34 (6.5)	208 (40.0)	124 (23.8)	81 (15.6)	73 (14.0)	278 (53.5)	242 (46.5)
The way company policies are put into practice	45 (8.7)	216 (41.5)	119 (22.9)	90 (17.3)	50 (9.6)	259 (49.8)	261 (50.2)
My pay and the amount of work I do	154 (29.6)	86 (16.5)	115 (22.1)	131 (25.2)	34 (6.5)	280 (53.8)	240 (46.2)
The chances for advancement on this job	120 (23.1)	102 (19.6)	119 (22.9)	143 (27.5)	36 (6.9)	298 (57.3)	222 (42.7)
The freedom to use my own judgment	68 (13.1)	165 (31.7)	95 (18.3)	98 (18.8)	94 (18.1)	287 (55.2)	233 (44.8)
The chance to try my own methods of doing the job	46 (8.8)	202 (38.8)	100 (19.2)	87 (16.7)	85 (16.3)	272 (52.3)	248 (47.7)
The working condition	40 (7.7)	228 (43.8)	77 (14.8)	61 (11.7)	114 (21.9)	252 (48.5)	268 (51.5)
The way my coworkers get along with each other	30 (5.8)	241 (46.3)	107 (20.6)	58 (11.2)	84 (16.2)	249 (47.9)	271 (52.1)
The praise I get for doing a good job	86 (16.5)	85 (16.3)	166 (31.9)	118 (22.7)	65 (12.5)	349 (67.1)	171 (32.9)
The feeling of accomplishment I get from the job	32 (6.2)	195 (37.5)	73 (14.0)	61 (11.7)	159 (30.6)	293 (56.3)	227 (43.7)
Magnitude of job satisfaction in private hospitals						212 (81.2)	49 (18.8)
Magnitude of job satisfaction in public hospitals						75 (29.0)	184 (71.0)
Overall magnitude of job satisfaction						287 (55.2)	233 (44.8)

NB: VD: very dissatisfied; D: dissatisfied; N: neutral; S: satisfied; VS: very satisfied; *n*: number of participants.

**Table 4:** Bivariable and multivariable analyses of factors associated with job satisfaction, public and private hospitals in Bahir Dar city, 2020 (*N* = 261).

Variables		Private hospitals (*N* = 261)				Public hospitals (*N* = 259)			
		Job satisfaction				Job satisfaction			
		Satisfied *N* (%)	Dissatisfied *N* (%)	COR (95% CI)	AOR (95% CI)	Satisfied *N* (%)	Dissatisfied *N* (%)	COR (95% CI)	AOR (95% CI)
Age	20-29	NA	NA	NA	NA	67	75	1	1
	≥30					8	109	12.17 (5.51, 26.85)	13.06 (4.83, 35.34)^∗∗^
Educational level	Diploma	69	28	1	1	18	55	1	1
	Degree	120	16	2.75 (1.41, 5.33)	2.40 (0.96, 5.97)	50	120	1.32 (0.70, 2.45)	1.43 (0.66, 3.10)
	Above degree	21	10	2.70 (0.74, 9.85)	0.84 (0.08, 8.27)	7	9	2.29 (0.46, 11.25)	2.24 (0.22, 22.82)
Monthly salary	<3653	67	17	1	1	NA	NA	NA	NA
	3653-5294	70	19	0.93 (0.44, 1.95)	1.35 (0.43, 4.19)				
	5295-7111	18	7	0.65 (0.23, 1.81)	0.24 (0.05, 1.06)				
	>7111	57	6	2.41 (0.88, 6.53)	1.11 (0.30, 3.99)				
Job description	Yes	203	39	5.78 (2.20, 15.18)	5.59 (1.28, 24.31)^∗^	68	135	3.52 (1.51, 8.21)	2.04 (0.71, 5.86)
	No	9	10	1	1	7	49	1	1
Short-term training	Yes	153	27	2.11 (1.11, 4.00)	0.68 (0.18, 2.54)	54	108	1.80 (1.01, 3.24)	0.69 (0.27, 1.74)
	No	59	22	1	1	21	76	1	1
Nature of work	Pleasant	49	34	0.36 (0.25, 0.51)	0.29 (0.10, 0.85)^∗^	36	136	0.57 (0.43, 0.75)	1.01 (0.43, 2.39)
	Unpleasant	163	15	1	1	39	48	1	1
Responsibility	Yes	170	21	5.39 (2.78, 10.44)	2.21 (0.80, 6.05)	59	102	2.96 (1.58, 5.54)	1.11 (0.43, 2.89)
	No	42	28	1	1	16	82	1	1
Work environment	Safe	174	24	4.76 (2.45, 9.24)	0.64 (0.20, 2.00)	43	69	2.23 (1.29, 3.87)	1.25 (0.52, 3.00)
	Unsafe	38	25	1	1	32	115	1	1
Coworker relationship	Good	192	37	3.11 (1.40, 6.92)	0.84 (0.24, 2.85)	56	120	1.57 (0.85, 2.87)	0.44 (0.16, 1.21)
	Poor	20	12	1	1	19	64	1	1
Autonomy	Yes	167	17	6.98 (3.55, 13.72)	3.00 (1.01, 8.95)^∗^	54	78	3.49 (1.94, 6.26)	1.70 (0.70, 4.10)
	No	45	32	1	1	21	106	1	1
Pay and benefit	Fair	NA	NA	NA	NA	17	28	1.63 (0.83, 3.20)	0.90 (0.32, 2.51)
	Unfair					58	156	1	1
Organizational policy and strategy	Comfortable	136	16	3.69 (1.90, 7.14)	0.46 (0.14, 1.44)	29	25	4.00 (2.13, 7.51)	1.42 (0.58, 3.49)
	Uncomfortable	76	33	1	1	46	159	1	1
Performance appraisal	Yes	40	7	5.46 (1.27, 23.51)	3.76 (0.88, 15.91)	21	35	1.65 (0.88, 3.09)	0.24 (0.09, 0.63)^∗∗^
	No	172	42	1	1	54	149	1	1
Supportive supervision	Adequate	153	13	7.18 (3.55, 14.50)	2.36 (0.75, 7.38)	44	42	4.79 (2.69, 8.53)	2.31 (0.89, 5.95)
	Inadequate	59	36	1	1	31	142	1	1
Education opportunity	Yes	NA	NA	NA	NA	17	23	2.05 (1.02, 4.11)	0.76 (0.24, 2.36)
	No					58	161	1	1
Reward and recognition	Yes	54	8	5.24 (1.56, 17.58)	4.10 (0.64, 26.02)	19	12	4.86 (2.21, 10.65)	4.09 (1.17, 14.29)^∗^
	No	158	41	1	1	56	172	1	1
Affective commitment	High	189	27	6.69 (3.28, 13.63)	3.01 (0.98, 9.23)	59	111	2.42 (1.29, 4.54)	0.92 (0.31, 2.69)
	Low	23	22	1	1	16	73	1	1
Normative commitment	High	141	13	5.49 (2.73, 11.03)	2.28 (0.68, 7.61)	42	48	3.60 (2.05, 6.33)	3.14 (1.30, 7.61)^∗^
	Low	71	36	1	1	33	136	1	1
Continuance commitment	High	NA	NA	NA	NA	22	36	1.70 (0.92, 3.16)	1.21 (0.55, 2.67)
	Low					53	148	1	1

^∗∗^*p* value < 0.01; ^∗^*p* value < 0.05. 1 = reference category; NA: not applicable.

**Table 5:** Bivariable and multivariable analyses of factors associated with overall job satisfaction, hospitals in Bahir Dar city, 2020 (*N* = 520).

Variables		Job satisfaction			
		Satisfied *N* (%)	Dissatisfied *N* (%)	COR (95% CI)	AOR (95% CI)
Work place	Public hospital	75	184	1	1
	Private hospital	212	49	10.61 (7.03, 16.01)	8.89 (5.14, 15.35)^∗∗^
Job description	Yes	271	174	5.74 (3.19, 10.30)	2.31 (0.90, 3.90)
	No	16	59	1	1
Short-term training	Yes	207	135	1.87 (1.30, 2.71)	0.65 (0.34, 1.21)
	No	80	98	1	1
Annual leave	Yes	197	136	1.56 (1.08, 2.23)	0.79 (0.45, 1.40)
	No	90	97	1	1
Nature of work	Pleasant	202	63	6.41 (4.36, 9.42)	1.82 (1.05, 3.15)^∗^
	Unpleasant	85	170	1	1
Work environment	Safe	217	83	4.66 (3.20, 6.79)	0.91 (0.49, 1.69)
	Unsafe	70	140	1	1
Coworker relationship	Good	248	257	3.07 (1.99, 4.75)	0.71 (0.36, 1.37)
	Poor	39	76	1	1
Autonomy	Yes	221	95	4.86 (3.32, 7.11)	2.37 (1.29, 4.33)^∗∗^
	No	66	138	1	1
Organizational policy and strategy	Comfortable	165	41	6.33 (4.19, 9.55)	0.86 (0.45, 1.64)
	Uncomfortable	122	192	1	1
Performance appraisal	Yes	61	37	1.42 (0.91, 2.24)	0.78 (0.42, 1.45)
	No	226	196	1	1
Supportive supervision	Adequate	197	55	7.08 (4.78, 10.48)	2.42 (1.33, 4.40)^∗∗^
	Inadequate	90	178	1	1
Reward and recognition	Yes	73	15	4.95 (2.75, 8.91)	3.04 (1.37, 6.75)^∗∗^
	No	214	218	1	1
Affective commitment	High	248	138	4.37 (2.85, 6.71)	1.62 (0.85, 3.09)
	Low	39	95	1	1
Normative commitment	High	183	61	4.96 (3.39, 7.24)	2.57 (1.48, 4.43)^∗∗^
	Low	104	172	1	1
Continuance commitment	High	135	61	2.50 (1.72, 3.63)	0.73 (0.43, 1.24)
	Low	152	172	1	1

^∗∗^Significant at *p* value < 0.01; ^∗^significant at *p* value < 0.05; 1 = reference category.

## Data Availability

All the data were included in the study, and data will be available upon a responsible request from the corresponding author.

## Abbreviations

AC: Affective commitment
AOR: Adjusted Odds Ratio
BSc: Bachelor of Science
CC: Continuance commitment
CI: Confidence interval
COR: Crude odds ratio
Dr.: Doctor
ETB: Ethiopian birr
HR: Human resource
HWs: Health workers
MPH: Master of Public Health
NC: Normative commitment
NGOs: Nongovernmental organizations
OC: Organizational commitment
PhD: Doctor of Philosophy
SDGs: Sustainable development goals
SHP: Sample health professional
SPSS: Statistical Package for the Social Sciences
THP: Total health professional
WHO: World Health Organization.
